# Influence of Industrial Environments on the Development of Respiratory Systems and Morphofunctional Features in Preadolescent Boys

**DOI:** 10.2478/v10078-011-0084-z

**Published:** 2011-12-25

**Authors:** Wioletta Dziubek, Zofia Ignasiak, Krystyna Rozek

**Affiliations:** 1 Department of Physiotherapy in Internal Diseases, University School of Physical Education, Wroclaw, Poland; 2 Department of Biostructure, University School of Physical Education, Wroclaw, Poland

**Keywords:** air pollution, lung development, motor abilities

## Abstract

The present study examines the differences between levels of selected structural and functional features of boys 11–13 years in age from regions with varying levels of air pollution, including an industrial and rural region. The sample consisted of 213 boys from the industrial region and 98 from the rural region. Somatic, respiratory parameters and motor abilities were evaluated in both groups. The analysis of respiratory parameters revealed significantly better development of respiratory systems in boys from the rural region. Additionally, motor abilities were also better developed in boys from the rural region.

## Introduction

The sequences of developmental changes are, to a large extent, genetically determined. The influence of the environment is particularly important in children because of the intensity of developmental processes and greater plasticity of younger bodies. Ecological hazards to the natural environment, including contamination of the soil, air and food with heavy metals (e.g., lead, mercury and cadmium), strongly affects the main functional features of the body and endangers proper development and health of the entire population, especially children (Cohen, 2001; Slawinska, 2000). For example, physical growth and sexual maturation are significantly delayed. Furthermore, elevated levels of lead in the blood are associated with irreversible mental and fine-motor developmental deficits (Bellinger, 2008; Cole and Winsler, 2010; Ignasiak et al., 2011; Lidsky and Schneider, 2003).

Exposure to a variety of toxicants and/or conditions during lung development has the potential to significantly affect the overall growth and function of the respiratory system. Toxic exposure to the lungs during development is likely to involve the disruption and/or alteration of a specific molecular signal or transcription factor, although to date, little information is available as to the precise impact of such exposures. However, the timing of exposure during development appears to be critical in the subsequent effects observed (Pinkerton and Joad, 2000).

Numerous studies suggest that the processes of cellular differentiation, branching morphogenesis, and overall lung growth can be affected by exposure to chemicals. The effects of exposure, however, are likely to be different for each period of development (Ji et al., 1998).

As a result of higher minute ventilation and levels of physical activity, children have greater exposure to air pollutants than adults (Plunkett et al., 1992). Children also spend more time outdoors than adults, resulting in increased exposure to outdoor air pollution (Wiley et al., 1991).

Lead is neurotoxic, especially during early childhood, potentially causing harm to a child’s brain, kidneys, bone marrow and other body systems. At high levels, lead can result in coma, convulsions and death (Chao and Kikano, 1993; US Environmental Protection Agency, 2001). The results of a recent study published by Gauderman et al. (2004) indicate that current levels of air pollution have chronic, adverse effects on lung development in children aged 10–18 years, leading to clinically significant deficits in attained FEV1 as children reach adulthood.

In addition to associations between air pollution and respiratory symptoms, asthma exacerbations, and asthma hospitalisations, recent studies have found links between air pollution and premature birth, infant mortality, deficits in lung growth, and possibly, development of asthma (Kim, 2004).

This study examines the differences between levels of selected structural and functional features of boys from two regions with differing degrees of air pollution - an industrial area with a high level of air pollution (the town of Polkowice) and a rural area with low levels of air pollution (the town of Jedlina Zdroj) - to evaluate the relationship between the functional parameters of the respiratory system and growth status and motor abilities of children 11–13 years old.

## Material and Methods

### Participants

The sample consisted of 311 boys, aged 11–13 years (age was computed from date of birth and date of examination). Of the total sample, 213 boys were from Polkowice, an industrial region with a high level of air pollution, and 98 boys were from Jedlina Zdroj, a rural region with low levels of air pollution. All tests were conducted in September 2002. The examinations were performed in the following order:

The first day of testing included the anthropometry and respiratory function tests. On the second day, the fitness tests were conducted, which included the plate tapping test, sit and reach, standing broad jump, handgrip, and shuttle run in that order (Eurofit 1993).

Stature, body mass, and skinfold thickness were measured according to the techniques described by Martin and Saller (1959). Stature was measured to the nearest millimetre using a Harpenden-type anthropometer. Body mass was measured to the nearest 100 grams using a balance scale. The thicknesses of three skinfolds were measured over the triceps, subscapular and abdominal areas using calipers (Holtain; Crymych) and added together to give the sum of skinfolds.

Only healthy children who had been qualified by a physician prior to testing were admitted to take part in the study. All tested children had lived in their cities from birth.

The study was approved by the ethics committee of the University School of Physical Education Wroclaw and the local school authorities.

### Family status

The social and economic situation was similar for both groups. There were no significant differences in the education level of parents (approximately 75% of parents had a vocational education), cigarette use or unemployment.

### The study location

The regions of Polkowice and Jedlina Zdroj were selected after an analysis of the air pollution data from the Lower Silesia was conducted. We selected contrasting areas with very high and very low air pollution levels.

The town of Polkowice is situated in the Lower Silesia, located in southwestern Poland, in the Legnica-Glogow Copper Mine District at 150 m above sea level.

Recent efforts have been made to reduce the emission of lead and other pollutants in the Legnica- Glogow Copper Mining District, but specific data for the region are limited. A report from a company operating smelters (KGHM Polska Miedz S.A.) indicated a reduction in lead emissions by 99% between 1985–2007 (www.kghm.pl). Particulate emissions in the Legnica-Glogow Region were greater than 3000 tons per year in 1995, 1996 and 1997, and varied approximately 2000 tons per year from 2002 through 2006 and 1400 tons per year in 2007 (Statistical Yearbook, 1996; Statistical Yearbook of the Regions – Poland, 2008).

National data for Poland, including the southwestern region, noted an increase in lead levels from 1990 to 1993, followed by a rapid decline until 1998 and a slight decline until 2007. Lead emissions in Poland have declined from 647, 5 tons/year in 2000 to 573, 4 tons/year in 2007 (Environment, 2009).

The outflow of lead through rivers leading to the Baltic Sea was also reduced from 124,7 tons per year in 1995 to 68,9 tons per year in 2007 (Environment, 2009). Although emissions have seemingly declined, it should be noted that lead is also in other heavy metals (e.g., Cu and Cd) and toxic compounds that have been emitted in the past remain in the soil and plants. Furthermore, the soil self-cleansing process takes many years in contrast to the relatively short self-cleansing times of air (a few days) and water (a few years).

The air contamination data are presented in Tables 1–4 (Report on the state of the environment of Lower Silesia, 1998–2003). The reports indicate the emissions of air pollutions per year. Mines, smelteries, foundries and plants associated with the copper industry generate large amounts of industrial waste, which contaminate the air and soil. Studies of the crops and soil in this region indicate a high level of contamination compared with other regions of Poland (Ditchen-Rynarzewska, 1992; Ignasiak et al., 2011).

The air pollution data from Polkowice and Jedlina Zdroj areas are presented in Tables 1–4.

The town of Jedlina Zdroj is located in a rural region with a mountain climate at ca. 500 m above sea level. Mineral water sources (hydrocarbon-calcium-magnesium-sodium sorrels) have existed in Jedlina Zdroj since the 16^th^ century, and the town has become a health resort. Recently, the spa has helped to treat respiratory system diseases, such as bronchial asthma, chronic obstructive pulmonary disease, chronic sinusitis and pneumoconiosis (Report on the state of the environment of Lower Silesia, 1998–2003).

The difference in altitude above sea level between Polkowice (150 m) and Jedlina Zdroj (500 m) is relatively small and according to published studies (Weitz et al., 2002), should not have a significant influence on the development of the respiratory system.

### Lung-Function Tests

Evaluation of lung function was performed using a commercial spirometer (Flowscreen, Jaeger). The following respiratory parameters were chosen for analysis: vital capacity (VC), forced expiratory volume in 1 s (FEV_1_), Tiffeneau-index (FEV_1_%VC), peak expiratory flow (PEF), maximal expiratory flow rate at 50% of FVC (MEF_50_) and maximal voluntary ventilation (MVV). The spirometric testing was conducted only in the sitting position. Each subject was asked to perform three satisfactory blows, defined as FVC and FEV_1_ agreeing within 5%, FEV_1_ extrapolation volume less than 100 ml or 5% of FVC, less than 50 ml expired in the final 2 s, and forced expiratory time exceeding 3 s. The best of the three blows by each child was chosen by the spirometer program, according to the guidelines of the American Thoracic Society (ATS) modified for children (American Thoracic Society, 1978; American Thoracic Society, 1996). Volume and gas calibrations were performed before each test with a 1-L syringe (3% variability was acceptable), and the results were corrected to BTPS conditions. The recommended reference values of the European Coal and Steel Community (ECSC) gave predictions for lung variables in children (Quanjer et al., 1993; Quanjer et al., 1995). A trained person performed the spirometric testing in all subjects.

### Motor Abilities Tests

Motor abilities were measured with selected European Personal Fitness Tests in the following order: plate tapping test, sit and reach, standing broad jump, handgrip, and shuttle run (Eurofit 1993).

All tests were performed in a gym. A non-slip surface and sport shoes were used for the running and jumping tests. The participants rested between each test. The battery of tests included the following:

-Plate tapping test, which measured the speed of upper limb movements. Participants were asked to pass, as quickly and as many times as possible, a plastic disc held by one hand over to the other, with the disc touching the flat surface of a table.
-Sit-and-reach test, which measured flexibility and included reaching as far as possible from a sitting position.
-Standing broad jump test, which measured explosive strength by jumping for a distance from a standing start.
-Handgrip test to measure static strength. This was achieved by squeezing a calibrated hydraulic hand dynamometer (Jamar) as forcefully as possible with the dominant hand.
-10 × 5 m shuttle run test, which measured running speed and agility by having participants run as quickly as possible between posts that were 5 m apart.

### Statistical Analysis

Statistical analyses were performed using STATISTICA 6.0 software. The significance of average value differences of individual somatic features, respiratory parameters and fitness tests between age groups was calculated with an ANOVA variance one-way analysis.

## Results

The baseline anthropometric characteristics and study results are presented in Table 5. While analysing some basic measurements of somatic development, it should be taken into consideration that only 11-year-old boys from the rural, less polluted region are significantly higher (p<0,05) than those from the polluted region. There were no significant differences in age, body mass, BMI and sum of skinfolds between boys from the two regions. The mean values of the spirometric parameters between children, however, were significantly different.

Lung function parameters were expressed as the per cent of predicted values (Table 6). Mild restrictive and obstructive-type abnormalities were identified in less than 10% of boys from the more polluted area only and increased with age. Boys from the rural, less polluted area did not have any respiratory function abnormalities, and their respiratory parameters were significantly higher compared with boys from the polluted area (Table 6).

The motor ability traits were generally lower in boys from the polluted region. However, the results of the plate tapping test were similar in both groups, and only the 11-year-old boys from the polluted region obtained significantly higher values in the sit-and-reach test (Table 5).

## Discussion

The participants in this study are at the beginning of puberty, an important phase in their lives, which is characterised by a considerable increase of somatic parameters and intensive changes of functional traits, connected with a major hormonal transformation. The changes result in the complete biological maturity of the body.

Somatic development in both communities was similar. Only the body height of the 11-year-old boys from the rural spa region was significantly higher. Siniarska et al. (1992) reported similar results, suggesting that somatic features are more resistant to environmental pollution than more labile functional features. Some authors, however, seem to suggest that even low heavy metal exposure may affect stature growth (Bellinger, 1998; Schell and Denham, 2003; Schell and Knutsen, 2002; Vivoli et al., 1993).

Environmental pollution associated with urbanisation and industrialisation has significant influence on the development and functioning of a human being. It can impact both the structure and the function of an organism.

As Slawinska (2000) maintains, the comparison of somatic development and physical efficiency indices of children and teenagers from clean and polluted regions is ambiguous. Some authors observe tendencies towards lower body mass and height parameters as well as a delay in puberty in children from industrial regions (Mleczko and Ambrozy, 1999; Silbergeld, 1997). Other researchers have not reported any differences in morphological structure. It seems probable, however, that children developing in highly polluted regions may have reduced physical efficiency (Mleczko and Ambrozy, 1999).

The results of this study confirm that air pollution affected lung function in preadolescent children. We have found that the respiratory function tests were significantly lower among boys living in the region with higher pollution levels.

The literature provides reports about the negative influence of air pollution on living organisms. Small dust particles may penetrate small bronchioles and alveoli, disturb the gas exchange and impair the abilities of organisms (Ignasiak et al., 2011; Plopper and Fanucchi, 2000).

Despite several programs aimed to improve its natural environment (Ignasiak et al., 2011; Slawinska, 2000), the Legnica-Glogow Copper Mine District has been recognised as a highly polluted region (Report on the state of the environment of Lower Silesia, 1998–2003). Official information implies that this region is one of the most contaminated areas in Poland. The environmental reports are supplemented by excessively unfavourable data regarding morbidity and mortality in this region (Report on the state of the environment of Lower Silesia, 1998–2003).

Our results indicate that the measured parameters were significantly better in children from the rural area and support the thesis that a polluted environment negatively affects a developing respiratory system.

Longitudinal studies involving serial evaluations of lung function in the same individual over shorter or longer periods of time in children may serve as a sensitive indicator of children’s current pulmonary health status and detect any possible changes caused by a hazardous environment (Dockery and Brunekreef, 1996). The American Thoracic Society (ATS) defined such changes in children as explicit markers of the adverse health effects of air pollution indicated by the “failure to maintain their predicted lung function curve in children” (American Thoracic Society, 1985).

Interpretation of pulmonary function development in children in terms of environmental hazards is difficult given the strong variations within a child’s stage of growth. In cross-sectional analyses, Wang et al. (1993) have shown that a child’s pulmonary function experiences a linear increase with age and height until the adolescent’s growth spurt, which occurs at approximately 10 years of age in girls and 12 years of age in boys.

Several studies have shown the negative adverse effects of air pollution on pulmonary ventilation (Gauderman et al., 2000; 2002; Horak et al., 2002; Plopper and Fanucchi, 2000; Sunyer, 2001). These observations prove that air pollution leads to chronic obstructive pulmonary disease and related decrease in FEV_1_. The relationship between pollution level (PM_10_) and degree of impairment of pulmonary ventilation was also noted (Meyer et al., 2003; Rojas-Martinez, 2007).

We have found significantly lower respiratory parameters in the participants from the polluted region. Moreover, ventilation disorders were also observed in a small percentage of boys. These results may reflect a negative influence of environmental pollution on children.

Our research confirms the thesis of ambiguous results with reference to physical efficiency. We have observed significantly higher flexibility parameters in boys from the polluted region but excessively higher speed abilities and lower extremities explosive force in boys from the rural region. Other measured abilities were similar in both studied groups.

Boys from the rural region had significantly better results for the physical efficiency tests (e.g., shuttle run, broad jump, and handgrip) compared with boys from the industrial region. This result can be partially explained by the fact that children from the rural region of Jedlina Zdroj engage in more spontaneous physical activity related to mountainous terrain. In addition to well-organised physical education at schools, spontaneous physical activity in a natural environment is a fundamental stimulus of motor development in early stages of ontogenesis (Mleczko and Ambrozy, 1999).

Differences in the development of motor abilities in children from varying geographical areas have not been sufficiently studied. Beunen et al. (1992) examined physical efficiency in children with differing amounts of physical education per week at school and found no significant differences.

In our study, the level of static muscle strength and flexibility was similar in both groups. The correlation of motor function tests with functional features of the respiratory system was better in the group from the rural region. A strong relationship was observed between the functional features of the respiratory system as well as both the static strength of hand muscles (similarly to their peers from polluted region) and the speed of arm movement. It can be assumed, then, that the relationship between the static strength of hand muscles and the functional features of the respiratory system is the result of the muscular strength of the body, which undoubtedly influences breathing functions.

## Conclusions

1. Anthropometric parameters were similar in both of the examined groups, with the exception of the significantly higher height of the 11-year-olds from the rural spa region.
2. Analysis of respiratory parameters revealed significantly higher levels of development of the respiratory system in boys from the rural region compared with the boys from the polluted region.
3. There was a slightly higher incidence of ventilation disorders in boys from the polluted region.
4. Motor abilities were better developed in boys from the rural region.

## Figures and Tables

**Table 1:** Permissible concentrations of air pollutants for Poland

***Pollutant***	***Concentration***	***Averaging period***	***Permitted exceedences each year***
Cuprum [μ/m^3^]	0,6 μg/m3	1 year	n/a
PM10	50 μg/m3	1 year	n/a
Lead (Pb)	0,5 μg/m3	1 year	n/a

**Table 2:** Air pollution in Polkowice per year

***Pollutant***	***Concentration***	***Averaging period***	***Permitted exceedences each year***
***1997***			
Cuprum [μ/m^3^]	0,09 μg/m3	1 year	n/a
PM10	17 μg/m3	1 year	n/a
Lead (Pb)	0,13 μg/m3	1 year	n/a
**1998**			
Cuprum [μ/m^3^]	0,06 μg/m3	1 year	n/a
PM10	20 μg/m3	1 year	n/a
Lead (Pb)	0,09 μg/m3	1 year	n/a
**1999**			
Cuprum [μ/m^3^]	0,08 μg/m3	1 year	n/a
PM10	29 μg/m3	1 year	n/a
Lead (Pb)	0,17 μg/m3	1 year	n/a
**2000**			
Cuprum [μ/m^3^]	0,02 μg/m3	1 year	n/a
PM10	11 μg/m3	1 year	n/a
Lead (Pb)	0,01 μg/m3	1 year	n/a
**2001**			
Cuprum [μ/m^3^]	−	1 year n/a	
PM10	25 μg/m3	1 year	n/a
Lead (Pb)	−	1 year	n/a
**2002**			
Cuprum [μ/m^3^]	−	1 year n/a	
PM10	25,5 μg/m3	1 year	n/a
Lead (Pb)	−	1 year	n/a

**Table 3:** Permissible concentrations of air pollutants in the Spa Conservation Area

***Pollutant***	***Concentration***	***Averaging period***	***Permitted exceedences each year***
Cuprum [μ/m^3^]	0,6 μg/m3	1 year	n/a
PM10	40 μg/m3	1 year	n/a
Lead (Pb)	0,35 μg/m3	1 year	n/a

**Table 4:** Air pollution in Jedlina Zdroj per year

***Pollutant***	***Concentration***	***Averaging period***	***Permitted exceedences each year***
***1997***			
Cuprum [μ/m^3^]	−	1 year	n/a
PM10	7 μg/m3	1 year	n/a
Lead (Pb)	0,05 μg/m3	1 year	n/a
**1998**			
Cuprum [μ/m^3^]	−	1 year	n/a
PM10	7 μg/m3	1 year	n/a
Lead (Pb)	0,07 μg/m3	1 year	n/a
**1999**			
Cuprum [μ/m^3^]	−	1 year	n/a
PM10	10 μg/m3	1 year	n/a
Lead (Pb)	0,03 μg/m3	1 year	n/a
**2000**			
Cuprum [μ/m^3^]	−	1 year	n/a
PM10	9 μg/m3	1 year	n/a
Lead (Pb)	0,002 μg/m3	1 year	n/a
**2001**			
Cuprum [μ/m^3^]	−	1 year	n/a
PM10	11 μg/m3	1 year	n/a
Lead (Pb)	−	1 year	n/a
**2002**			
Cuprum [μ/m^3^]	−	1 year	n/a
PM10	15,4 μg/m3	1 year	n/a
Lead (Pb)	−	1 year	n/a

**Table 5:** Somatic, respiratory and motor parameters of boys (11–13 years in age) from the polluted and rural regions

	Polluted region			Rural region		

Age groups [yrs]	11,0±0,3	12,0±0,3	13,0±0,3	10,9±0,3	11,9±0,3	13,0±0,2
Number [n]	79	58	76	38	30	30

Height [cm]	144,1±6,7	149,9±7,3	158,4±8,4	147,6±7,8*	150,6±7,6	156,7±9,9
Weight [kg]	37,1±9,0	39,7±9,6	48,4±11,3	39,2±7,7	41,0±8,6	45,2±9,2
BMI [kg/m^2^]	17,7±3,0	17,5±3,1	19,2±3,6	17,9±2,7	17,9±2,8	18,2±1,7
Sum of skinfold thickness [mm]	29,9±14,2	28,4±14,9	30,6±14,3	29,2±14,7	28,7±13,7	27,4±13,6
VC [l]	2,7±0,6	2,9±0,5	3,4±0,7	3,2±0,6***	3,4±0,6**	3,8±0,9**
FEV1 [l]	2,4±0,4	2,6±0,4	3,0±0,7	2,8±0,6***	3,0±0,5***	3,5±0,7*
PEF [l/s]	4,6±0,9	5,0±1,1	5,8±1,3	5,6±1,0***	5,8±1,0**	6,9±1,7***
MEF50 [l/s]	3,2±0,6	3,6±0,7	3,9±1,1	3,9±0,8***	4,15±0,8**	4,5±1,2*
MVV [l/min]	66,7±12,5	74,8±15,7	86,5±19,4	78,8±18,2***	83,1±13,7*	95,9±19,1*
Plate tapping [s]	14,1±2,0	12,9±1,5	12,1±1,4	14,1±1,8	13,6±1,7	12,1±1,4
Sit-and-reach [cm]	19,9±5,9**	18,1±7,3	16,3±9,6	15,8±9,6	14,8±9,2	15,1±10,8
Standing broad jump [m]	1,34±0,2	1,40±0,2	1,55±0,2	1,51±0,2***	1,57±0,2**	1,73±0,3**
Handgrip [kG]	19,8±3,6	22,3±4,6	26,7±5,8	21,1±3,8*	25,0±4,9*	27,9±7,7*
Shuttle run [s]	21,8±1,8	21,2±1,8	20,5±1,7	18,9±1,9***	18,9±1,5***	19,0±1,8***

Values are expressed as the mean ±SD

*** Significant difference between groups: p<0,000

** Significant difference between groups: p<0,001

* Significant difference between groups: p<0,05

**Table 6:** Lung function parameters expressed as a percentage of predicted values in boys from both regions

	Polluted region			Rural region		

Years	11	12	13	11	12	13
VC [%]	101,7±12,2	99,4±13,4	97,7±14,2	111,1±16,6*	112,6±12,7*	111,9±17,9*
FEV1 [%]	110,2±14,2	107,6±14,1	106,2±16,5	120,9±23,4*	121,7±17,1*	118,2±15,5*
FEV1%VC	88,5±6,5	88,7±6,2	88,6±7,3	88,7±4,7*	88,8±6,1*	88,1±5,6*
PEF [%]	94,7±17,6	94,2±19,0	95,1±18,1	108,9±16,5*	108,1±15,8*	116,6±20,3*
MVV [%]	150,2±34,6	151,4±28,8	144,8±34,8	166,9±32,7*	167,1±26,9*	170,4±26,3*
Obstructive disorders [%]	6,3	8,62	9,21	0	0	0
Restriction disorders [%]	2,6	3,5	5,3	0	0	0

Values are expressed as the mean ±SD

* Significant difference between groups: p<0,05
